# Straight and tilted implants for supporting screw-retained full-arch dental prostheses in atrophic maxillae: A 2-year prospective study

**DOI:** 10.4317/medoral.22459

**Published:** 2018-11-21

**Authors:** Manuel Menéndez-Collar, Maria-Angeles Serrera-Figallo, Pilar Hita-Iglesias, Raquel Castillo-Oyagüe, Juan-Carlos Casar-Espinosa, Aida Gutiérrez-Corrales, José-Luis Gutiérrez-Perez, Daniel Torres-Lagares

**Affiliations:** 1 Department of Stomatology, Faculty of Dentistry, University of Seville (US), C/Avicena, s/n, 41009, Seville, Spain; 2 Department of Oral & Maxillofacial Surgery, University of Michigan School of Dentistry, Ann Arbor, Mich; 3 Department of Buccofacial Prostheses, Faculty of Dentistry, Complutense University of Madrid (U.C.M.), Pza. Ramón y Cajal, s/n, 28040, Madrid, Spain

## Abstract

**Background:**

To evaluate, over a 2-year period, the treatment outcomes for maxillary full-arch fixed dental prostheses (FDPs) supported by a combination of both tilted and axially-placed implants and to compare the marginal bone loss (MBL) and implant survival rates (SR) between tilted and axial implants.

**Material and Methods:**

A retrospective study has been carried out. Thirty-two patients (16 males and 16 females) treated with maxillary full-arch FDPs were included in this retrospective study. A total of 187 implants were inserted to rehabilitate the fully edentulous maxillary arches: 36% of them were tilted (T group, n = 68) and the remaining 64% were axially placed (A group, n = 119). From the total, 28% of the implants (n=53) were immediately loaded with screw-retained provisional acrylic restorations, whereas 72% underwent conventional delayed prosthetic loading 6 months post-operatively. Definitive restorations were hybrid implant prostheses (metal framework covered with high-density acrylic resin) and metal-ceramic screw-retained implant prostheses, and were placed 6 months after surgery. Such definitive restorations were checked for proper function and aesthetics every three months for two years. Peri-implant marginal bone levels were assessed by digital radiographs immediately after surgery and MBL was assessed at definitive implant loading (baseline) and 2 years afterwards.

**Results:**

The 2-year implant SR were 100% for axially placed implants and 98.5% for tilted implants. No significant differences were found amongst the A and T implant groups. Marginal bone loss measured at 2 years after definitive prosthetic loading was of -0.73 ± 0.72 mm (maximum MBL of 1.43 mm) for axially positioned implants vs. –0.51 ± 0.92 mm for tilted implants (maximum bone 1.45 mm). Differences in MBL were statistically significant when comparing immediately and delayed loaded implants.

**Conclusions:**

Based on the results of this retrospective clinical study, full-arch fixed prostheses supported by a combination of both tilted and axially placed implants may be considered a predictable and viable treatment modality for the prosthetic rehabilitation of the completely edentulous maxilla.

** Key words:**Tilted implants, full-arch dental prostheses, atrophic maxillae, marginal bone level.

## Introduction

Dental implants constitute a complex and multifactorial treatment in the reconstruction of the edentulous maxilla that requires the proficiency and collaboration of the surgeon and the restorative/prosthodontic dentist. Much of the challenge in the reconstruction of the atrophic maxilla lies on the presence of crestal bone resorption and anatomical limitations such as the maxillary sinus. These two often lead to bone augmentation procedures associated with high cost an increased risk of morbidity and poor patient acceptance. The toolbox for oral and maxillofacial surgeons offers a variety of clinical techniques and concepts to eliminate the need for bone augmentation procedures in the severely resorbed and atrophic maxillae.

The use of cantilevered implant-supported fixed dental prostheses (FDPs) has been suggested as an alternative in posterior regions where placing additional implants represents a challenge due to lack of bone height and/or crest width (1). Distal cantilevers may reduce the healing time and treatment costs. However, the biomechanical performance of implant-supported rehabilitations with cantilevers has been associated with low survival rates and frequent biologic and technical complications (2,3). In addition, the survival rates for this type of treatment with distal extensions longer than 15 mm are lower than with shorter cantilevers (4).

Short implants (8 mm or less) could be a possible option, but a minimum amount of at least 7 mm of vertical bone height must exist (5). Moreover, adequate bone quality is critical for achieving success with short implants (2). The use of pterygoig and zygomatic implants have been proposed as an alternative to bone grafting procedures in the rehabilitation of the posterior atrophic maxilla (6) with cumulative success rate of zygomatic implants ranging from 74% to 99% (7). However, the placement of either type of implant is very technique-sensitive and invariably presents with a high rate of biological and technical complications.

The use of tilted implants (placed distally, either parallel to the anterior wall of the maxillary sinus or mesial to the mental foramen nerve) has been proposed by several authors within the past decade as a viable treatment option for the prosthetic rehabilitation of the severely atrophic posterior jaws (8). Their advantages include a greater anchorage of the implant to the cortical plate as well as the possibility to avoid vital anatomical structures. Current findings from clinical studies comparing both tilted and axially placed implants show similar success rates (SR), and marginal bone loss (MBL) for either type of implant (6). Nevertheless, more long-term clinical data is needed to further support its use as a predictable treatment modality in modern implant dentistry.

## Material and Methods

-Study protocol and participants

The present study was conducted according to the Code of Ethics of the World Medical Association (Declaration of Helsinki); the Spanish Law 14/2007 for Biomedical Research; and the Uniform Requirements for manuscripts submitted to Biomedical journals. The approval of the Ethics Committee of the University of Seville (U.S., Spain), was obtained once the ethical board completed an independent review of the research protocol and the inform consent.

Patients presenting with complete maxillary edentulism and severe posterior atrophy were recruited and operated on between January 2007 and December 2012 at a private practice office in Cordoba, Spain. A total of 187 implants were placed by a surgical team of two Oral Surgeons from the University of Seville (U.S., Spain). Definitive restorations were designed and fabricated in conjuction with an expert prosthodontist (Complutense University of Madrid, U.C.M., Spain).

In this study, we followed the definition of angled implant proposed by Aparicio *et al.* (8) to differentiate tilted from axially placed implants. Thus, all implants placed with an inclination equal or greater than 15 degrees in relation to the occlusal plane (whether in a mesio-distal, disto-mesial and/or bucco-palatal direction) fell into the category of tilted implants (9).

The inclusion criteria were: systemically healthy patients (ASA classification I or II), fully edentulous patients aged 18 years or older; patients who declined wearing complete removable dentures (CRD); residual alveolar bone lesser than 8 mm measured from the floor of the maxillary sinus to the alveolar crest; patients who voluntarily signed the informed written consent to participate in the study; and patients who were compliant with clinical and radiographic follow-up appointments. The exclusion criteria were the following: presence of active infection or swelling at the implant site; patients with severe illnesses such as uncontrolled diabetes, autoimmune disorders and coagulopathies; patients who had undergone radiation therapy of the head or neck in the past 12 months; pregnant women; inability or unwillingness to maintain a good level of oral hygiene; and incapability or refusal to return for follow-up visits.

Two different implant systems were used: a) Nobel Speedy Groovy R.P., (Nobel Biocare AB, Göteborg, Sweden) and (b) Biomet 3i (Dental Ibérica, Barcelona, Spain) In addition, a total of four models of Biomet 3i implants were used, including Osseotite Implant, Full Osseotite Implant, NanoTite Implant and NT Osseotite tapered Implant.

-Preoperative and Surgical protocols

Prior to implant surgery, patients were distributed into 2 groups depending on the postoperative implant loading protocol. Patients in group A received a temporary wrought-wire clasped acrylic removable complete denture (RCD) and patients in group B underwent immediate screw-retained implant loading. Only those implants that achieved an insertion torque of at least 40 Ncm were load immediately.

All patients underwent the surgical procedure with loco-regional anesthesia. Articaine 4% 1:100000 epinephrine (Artinibsa, Laboratorio Inibsa, Barcelona, Spain) or Mepivacaine 2% (Scandinibsa, Laboratorio Inibsa, Barcelona, Spain) if the vasoconstrictor was contraindicated, were used. The day of the implant surgery all patients received 6 mg betamethasone I.M. (Celestone 2 ml, Laboratorio Merck Sharp & Dohe, Madrid, Spain) and 2g amoxicillin/125 mg clavulanic acid or 500 mg Azitromizin (Azitromicina Stada 500mg, Laboratorio Stada. S/L, Barcelona, Spain.) if allergic, 1 hour preoperatively. Such antibiotic regime was postoperatively continued for 7 days.

After adequate anesthesia was obtained using block and infiltration techniques, the surgical field was prepared following the standard one-stage non-submerged protocol for implant surgery. A full-thickness mucoperiosteal flap was reflected by a mid-crestal incision made slightly palatal in combination with a single vertical releasing incision placed onto the maxillary tuberosity. The envelope flap was retracted and the underlying buccal bone was then exposed at the level of the maxillary sinus wall. Using the imaging information from the preoperative radiographic study, a lance drill was used to locate the maxillary sinus and a Nabers probe (Nabers PQ2n, Hu Friedi Mfg. Co., LLC, USA) was used to examine the anterior sinus wall. First, the most distal implants (T) were placed following the anatomy of the aforementioned wall, with an angulation between 20° and 45° in relation to the occlusal plane. Attention was paid towards placing the implant platform as distal as possible. Second, the axial implants (A) were anteriorly placed and all fixations were then evaluated for primary stability thus concluding the surgical procedure. Two different healing abutments were used for each type of implant. Multiunit Abutments (MUA) low profile, either 30° or 17° pre-angulation were chosen for the T implants, whereas non-angulated UCLA abutments were connected to the A implants. A torque of 35 N/cm was applied to screw the abutments in all cases.

The surgical flap was repositioned onto the maxillary bone and sutured in place with resorbable single stitches (Monocril 5/0, Johnson & Johnson P.P., S.A., Madrid, Spain).

Patients were prescribed 10 ml rinses of 0.12% chlorhexidine twice daily for 14 days, as well as 25 mg dexketoprofen T.I.D. for 5 days (Enantyum 25 mg, Laboratio Menarini, Barcelona, Spain). Postoperative instructions were given to the patients and follow-up appointments were scheduled on a weekly basis for the subsequent month.

-Restorative procedures 

Definitive screw-retained implant-supported FDPs were delivered six months after implant placement (vacuum-cast Co-Cr frameworks coated with feldspathic ceramic; i.e., Heraenium Co-Cr and Hera Ceram; Heraeus-Kulzer, Wehrheim, Germany).

Appropriate transfer copings were used and a single-phase silicone impression technique with individual trays was taken (Imprint II, 3 M ESPE, Flexitime, Heraeus-Kulzer, Wehrheim, Germany). The final implant-supported FDPs (both metal-ceramic screw-retained and metal-resin hybrid prostheses) were fixed to their respective abutments tightening the screws to 30 N/cm. Both the static dental relationships (maximum intercuspation) and the dynamic occlusion (canine and anterior guidance) were checked. All prematurities and interferences were removed.

We considered the six-month postoperative mark the baseline point for all follow-up measurements.

-Implant success criteria

Following the criteria established by Albrektsson *et al.* in 1986, implant success findings at two-year follow-up included the presence of implant stability, no radiographic evidence of peri-implant pathology or signs or symptoms of infection, absence of pain and MBL not exceeding 2 mm (10).

-Follow up 

The FDPs were clinically checked for proper function and aesthetics every three months for two years. Intraoral digital radiographs (Schick CDR, Schick Technologies, Long Island City, NY, US) were obtained immediately after surgery, at the moment of definitive implant loading (baseline) and 24 months after baseline. Periapical radiographs were taken following a long-cone parallel technique with an occlusal template to assess the changes in marginal bone level over time.

In this study the marginal bone height/level was defined as the distance between the lower edge of the implant platform and the most coronal point of contact between the bone and the fixations (11). Measurements of marginal bone height were taken by the same blinded operator and measured in mm (rounded to the nearest 0.01 mm) at the mesial and distal aspects of each implant and a mean value was calculated for each implant and for each study group at baseline and after two years of definitive implant loading to the closest half thread using specific software (Schick CDR, Schick Technologies) Consequently, the variable MBL was defined as the average difference in marginal bone height recorded between the time of definitive implant loading and two years afterwards.

Information about possible modulators of MBL was also recorded and classified into two subcategories:

a) Patient-dependent (non-modifiable) variables: age, gender, smoking habits (smoker, former smoker, or non-smoker), medical condition (patients on medication or not), bone quality/type according to the Lekholm and Zarb classification (12), shape of the alveolar ridge according to the classification of Misch and Judy (13), and type of occluding dentition.

b) Surgical and/or rehabilitation-dependent (independent) variables: implant brand/model (mainly determined by the position of the implant platform), implant length, location (premolar or molar), and wearing or not a temporary prosthesis (acrylic removable partial denture, RPD).

-Statistical analysis

The statistical analyses were applied according to the requirements for the design of clinical trials in implant dentistry revised at the 8th European Workshop on Periodontology, (14) as well as the recommendations of Hannigan and Lynch for oral and dental research (15). Data were processed using the Statistical Package for the Social Sciences (software v.22) (SPSS/PC+, Inc.; Chicago, IL, USA), applying the cut-off level for statistical significance at α = 0.05 (16,17).

Mean and standard deviations (SD) were calculated for all of the study variables (16). The intra-examiner error was determined by the Kappa test (17). The normal data distribution was confirmed by the Kolmogorov-Smirnov test and the homogeneity of variances was verified according to the Levene’s test (15).

Survival rate comparisons between S and T implants were conducted using the X2 and the Fisher’s exact tests (18).

Differences in MBL between S and T implants were compared using a two-tailed Student’s t-test (15). To further evaluate the statistical significance of possible modulating factors on MBL, the Student’s t-test was used for bi-categorical variables, whereas the ANOVA with Bonferroni’s post-hoc tests were run to assess the differences in multi-categorical factors (17).

## Results

-Descriptive statistics

Thirty-two patients (16 males and 16 females; mean age: 55.25 ± 9.89 years) were included in this retrospective study. A total of 187 implants: 98 Biomet 3i (52%) and 89 Nobel Biocare (48%) were placed in completely-healed sites. A hundred and nineteen implants (64%) were placed axially (Group A), and 68 (36%) implants were tilted (Group T: mean angulation of 35.9 ± 8.3 degrees). One tilted fixation was lost two years after implant loading (resulting in a final sample size of n = 67 for the T group).

All study participants were treated with complete maxillary FDPs supported by a combination of S and T implants. Thirty implants (16%) were connected to metal-ceramic screw-retained prostheses and 157 implants (84%) supported hybrid prostheses.

The Kappa statistics showed a perfect intra-assessment coefficient of reliability (k =1).

Data regarding the distribution of the patients with reference to the dependent and modifiable variables are displayed in  Table 1,  Table 1 continue. The mean follow-up period for all groups was 24.0 ± 1.23 months.

**Table 1:**
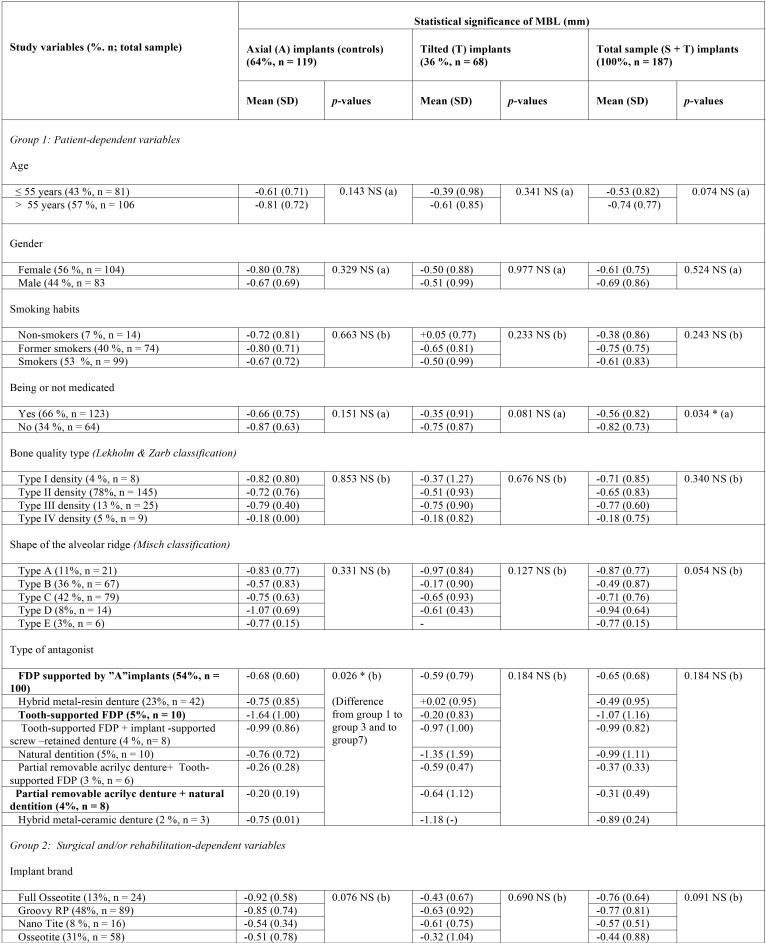
Influence of the study variables on the MBL (mm) of the tested groups (N = 187).

**Table 1 continue:**
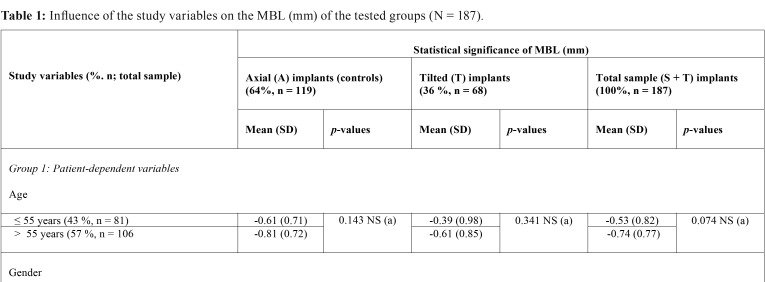
Influence of the study variables on the MBL (mm) of the tested groups (N = 187).

-Distribution of non-modifiable variables

Out of the 187 implants, 83 (44.4%) were inserted in males and 104 (55.6%) in females.

With regard to the type of bone in accordance to the Lekholm and Zarb classification (12), 8 implants (4%) were inserted in type I bone, 145 implants (78%) were inserted in type II bone, 25 implants (13%) were inserted in type III bone and 9 implants (5%), in type IV bone.

Ninety-nine implants (53%) were placed in smokers, 14 implants (7%) in non-smokers, and 74 implants (40%) were placed in former smokers.

Sixty-four implants (34%) were placed in healthy subjects and 123 implants were placed in patients with associated medical pathologies.

According to the alveolar ridge shape classification proposed by Misch and Judy (13), 21 implants (11%) were placed in type A alveolar ridge, 67 implants (37%) were placed in type B, 79 implants (42%) were placed in type C, 14 implants (8%) were placed in type D, and 6 implants (3%) were placed in type E alveolar ridge.

Concerning the antagonists, 99 implants (53%) opposed a combination of an implant-supported partial denture and natural teeth, 42 implants (23%) opposed implant overdentures, 10 implants (5.5%) opposed acrylic RPDs, 10 implants (5.5%) opposed natural dentition, 8 implants (4%) opposed a combination of natural teeth and an acrylic RPD, 8 implants (4%) opposed a combination of implant-supported and teeth-supported FDPs, 3 implants (2%) opposed metal-ceramic implant- supported dentures, and 6 implants (3%) opposed a combination of a teeth-supported FDP and an acrylic RPD.

Eighty-nine implants (48%) were NobelSpeedy Groovy, 24 implants (13%) were Biomet 3i Full OSSEOTITE, 16 implants (8%) were Biomet 3i NanoTite and 58 implants (31%) were Biomet 3i O OSSEOTITE.

Two implants (1%) were 8.5 mm in length, 10 (5%) were 10 mm, 17(9%) were 11.5 mm, 70 (37%) were 13 mm, and 88 (48%) were 15 mm in length.

Sixty implants (32%) were inserted in the incisors sites, 35 implants (19%) were inserted in the canines area, 52 implants (28%) were inserted in the premolars region, and 40 implants (21%) were placed in the molar sites.

Finally, 53 implants (28%) underwent temporary immediate loading with screw-retained prostheses, whereas 134 implants (72%) received a temporary removable acrylic denture followed by conventional delayed prosthetic loading 6 months postoperatively.

-Modulating factors of MBL

a) Effect of the study variables on the MBL for both A and T implant groups

The effect of possible modulating factors on the MBL is outlined in  Table 1,  Table 1 continue. A and T implants were not differentiated, as they were considered pieces of the same supporting combination for FDP maxillary restorations. Hence, the type of opposing dentition as well as the wear of a temporary RPD during the osseointegration period significantly affected the MBL ( Table 1,  Table 1 continue).

b) Influence of the study variables on the MBL of straight and tilted implants

The influence of the study variables on the peri-implant MBL for each group of implants (A and T) is displayed in  Table 1,  Table 1 continue. The patient-dependent variables assessed did not significantly affect the MBL for the T implant group ( Table 1,  Table 1 continue).

Wearing a temporary RPD during the osseointegration period (6 months post-operatively) was identified as a significant modulator or ‘predictor’ of MBL for A implants at two-years follow-up ( Table 1,  Table 1 continue); showing a statistically significant difference when compared to the MBL found in the T group (*p* = 0.001) ( Table 1,  Table 1 continue).

Comparison of marginal bone loss (MBL) and survival rates (SR)

Table 2 contains the values for mean (SD) marginal bone height and MBL registered at baseline and two years afterwards. No significant average differences in MBL were found between A and T groups at implant loading and at two years follow-up (*p* > 0.095) ( Table 2).

**Table 2:**
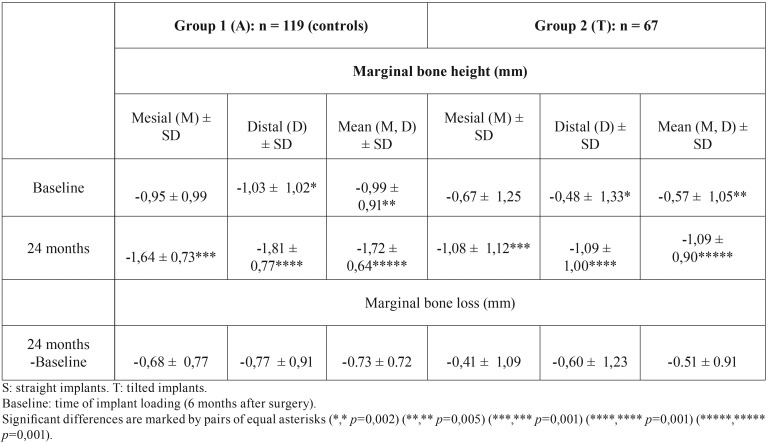
Radiographic marginal bone height at baseline and at 24 months follow-up, and marginal bone loss between both measuring times (N = 187 implants).

The SR was 98.5% for the A group (one implant failed at two-years follow-up) and of 100% for the T implants.

## Discussion

The rehabilitation of edentulous jaws with osseointegrated implants has been proven to be a predictable treatment over time (19). However, the implant rehabilitation of the edentulous atrophic maxilla still represents a clinical challenge due to anatomical limitations such as reduced bone volume particularly in the premolar–molar region (5).

Different techniques have been suggested to approach the rehabilitation of the edentulous maxilla. The use of distal cantilevers in the absence of posterior implants has been proposed with survival rates ranging between 50% to 100% (20). Romanos *et al.* assessed the clinical success of distal cantilevers of fixed full-arch prostheses in conjunction with immediate loading in a sample of 203 implants, thus obtaining an implant success rate of 94.5%, an implant survival rate of 97.5%, and a prosthetic survival rate of 96.7% at five years (21).

Malo *et al.* analysed the outcome of implant-supported FDPs with cantilevers after 5 years of prosthetic loading. Their study sample included 225 implants. The incidence of biological and mechanical complications in their investigation were 2.9% and 27.6%, respectively. Nonetheless, they registered a success rate of 99% and concluded that, despite the relatively high frequency of complications encountered, a fixed implant-supported partial rehabilitation with cantilever may be a viable treatment option (3).

Kim *et al.* (22) investigated the biological and technical success outcomes of implant-supported FDPs with and without cantilevers after a minimum of one year of prosthetic loading. A total of 28 cantilever FDPs (cFDP) supported by 132 implants were compared with 144 non-cantilever FPDs (ncFDPs) supported by 203 implants. Implant survival and success rates were 96.7% and 87.9% for implant supported cFDPs; and 99.5%, and 92.6% for ncFDP. Their study findings demonstrated a higher MBL for the posterior implants in the cantilever group although no significant differences in overall bone loss between cFDPs and ncFDPs were found.

Other treatment option is the combination of maxillary sinus lift and bone augmentation/grafting procedures. Authors such as Del Fabbro *et al.* (23) and Chiapasco *et al.* (24) reported similar success rates (92.5%) with this technique, (which may yield different clinical outcomes depending on the surgical protocol). Possible complications include those related to the donor site in case of autogenous grafting, and to the sinus surgery itself (i.e., sinusitis, loss of the graft, and perforation of the sinus membrane, amongst others) (25).

The use of zygomatic implants and implants placed in the pterigomaxillary region to rehabilitate the atrophic maxilla has been supported in the literature by various authors with cumulative survival rates ranging from 98.2-99% (7). Some studies have reported a zygomatic success rate of 98.5% (26). Nevertheless, the incidence of both mechanical 44% and biological complications 87.3% with the use of zygomatic implants remains significantly high (27).

The placement of tilted implants offers both surgical and prosthodontic advantages. Actually, the combination of tilted and axial implants allows for the use of longer implants (thereby increasing the osseointegration surface); improves the primary stability by anchoring in more than one cortical layer; limits cantilever extensions by placing the implants more distal and with a more optimal load distribution over the dental arch; and avoids the use of bone grafts and sinus lift procedures (with the resulting reduction in technique morbidity) (28).

Nonetheless, it has been assumed that the use of tilted implants could negatively affect the treatment outcomes due to the presence of unfavorable forces applied to the peri-implant alveolar bone. In our study, tilting the distal implant did not affect marginal bone level changes after 24 months follow up. This data compares favorably to the results previously published by several authors (2).

In a study carried out by Hinze *et al.* (2) in 47 patients who underwent treatment with either mandibular or maxillary full-arch FDPs supported by two axially inclined and two tilted implants, the 1-year implant survival rates were 96.0% for axially positioned implants and 94.6% for tilted fixations; so that no significant differences were encountered among both types of implants.

Francetti (29) found similar results when analysing the clinical outcomes about the changes of peri-implant bone level around tilted and axial implants supporting full-arch fixed immediate rehabilitations up to 60 months of loading. No significant differences in marginal bone loss were identified between axial and tilted implants in both jaws concluding that the use of tilted implants in the immediate rehabilitation of fully edentulous jaws is safe and is not associated to a higher marginal bone loss as compared to axially placed implants.

Several studies have exclusively focused on the immediate implant function in the edentulous maxilla. Cavalli *et al.* reported a series of 34 immediately loaded full-arch maxillary FDPs supported by tilted and axial implants with a cumulative implant survival rate of 100%. They highlighted the importance of an effective recall program in order to early intercept and correct possible prosthetic and biologic complications and thus prevent implant failure (30). In the same line, Maló *et al.* (31) published a study of 32 patients with the placement of 128 dental implants (64 angled and 64 axial), the reported success rate being 95.3% and 100%, respectively. The marginal bone loss was 0.9 mm on average, with no differences between the tilted and axial implants.

In the metaanalysis by Ata-Ali *et al.* (28), no differences were found when comparing success rates for tilted and axial implants. These results are in accordance with those published later on by Del Fabbro *et al.* (23), who conducted a systematic review to compare the crestal bone level change around axially placed vs. tilted implants supporting fixed prosthetic reconstructions for the rehabilitation of partially and fully edentulous jaws, after at least 1 year of loading. Tilting of the implants did not induce significant alteration in crestal bone level. Their results demonstrated that tilting of the implants does not induce significant alterations in crestal bone level changes as compared to conventional axial placement after 1 year of loading.

In the present study both A and T implants were considered the analytical units rather than the randomisation units (i.e., the patients); subsequently widening the confidence intervals and reinforcing the homogeneity between tilted and axial implants.

No statistically significant differences were found in MBL at baseline and at 24 months when comparing A and T implant groups. These results are in line with those published by authors (2,23). In the metaanalysis published by Ata Ali *et al.* (28), out of the 13 studies reviewed (7 retrospectives and 6 prospective) only 4 of them showed different MBL for tilted and axial implants. However, the overall trend observed was the absence of significant differences in the clinical outcomes of tilted vs. axially placed implants.

Wearing provisional RPDs during the osseointegration period resulted in significantly higher MBL with respect to wearing temporary fixed restorations ( Table 1). This finding is in accordance with that of Rossetti *et al.* (32), who reported that the previous use of a removable prosthesis was a risk factor for resorption, with flabby tissues related to the severity of bone loss. Furthermore, the use of immediate loaded full-arch FDPs supported by anterior axial implants and distal tilted implants has shown encouraging clinical results for restoring edentulous jaws (2).

The use of tilted implants yields several clinical advantages including minimizing the cantilever length, thus allowing for optimal load distribution (2). Besides, the use of tilted implants facilitates the placement of longer fixations, which subsequently increases the surface area of bone-implant contact and implant primary stability by providing anchorage to more than one cortical (33).

Our preliminary data suggest that the rehabilitation of the edentulous atrophic maxilla using a combination of A and T implants is a viable and predictable treatment modality that offers a high implant and prosthesis survival rate. In addition, it eliminates the need for more complex treatment procedures such as bone grafting sinus augmentation or zygomatic implants; which are associated with high cost, increased risk of morbidity, and poor patient acceptance (6,8).

Finally, and within the limitations of this study, the following conclusions may be drawn: 1. Neither the implant survival rate nor the peri-implant marginal bone loss seems to be affected by the inclination of the implants with respect to the occlusal plane. 2. Full-arch fixed restorations supported by a combination of axial and tilted implants could be a viable treatment option for the atrophic maxilla. 3. The treatment predictability and clinical outcomes of axial and tilted implants seem to be comparable.
